# Synthesis, characterization and X-ray structural studies of four copper (II) complexes containing dinuclear paddle wheel structures

**DOI:** 10.1186/1752-153X-7-35

**Published:** 2013-02-21

**Authors:** Samson Jegan Jenniefer, Packianathan Thomas Muthiah

**Affiliations:** 1 School of Chemistry, Tiruchirappalli, Tamil Nadu, 620024, India

**Keywords:** Paddle wheel structures, Cu (II) complexes, Supramolecular architectures, Carboxylates, Crystal structure

## Abstract

**Background:**

Various dinuclear copper (II) complexes with octahedral geometry have been reported. The majority of these complexes contain N containing aromatic rings as axial ligands. There are also a few cases where the solvent used in the reaction occupies the axial position of the dinuclear copper (II) complex. This may occur by planned synthesis or some times by serendipity. Here we report some four copper (II) complexes containing solvent and or N containing heterocyclic ring as the axial ligand.

**Results:**

Four compounds, each containing dinuclear Copper (II) units (with the most robust, frequently occurring paddle wheel structures) were synthesized and characterised by single crystal X-ray diffraction and by IR spectroscopy. The compounds 1 & 2 have the general formula Cu_2_(RCOO) _4_(L)_2_ [(for (1) RCOO= 4-Chloro Benzoate, L= Isopropanol; for 2 RCOO= Benzoate, L= 2-Amino-4,6-dimethyl pyrimidine )] while 3 & 4 have the general formula, Cu_2_(RCOO) _4_(S)_2_ Cu_2_(RCOO) _4_(L)_2_ [RCOO=5-Chloro-thiophene-2-carboxylate L= 2-Amino-4,6-dimethyl pyrimidine, for 3 S= ethanol; for 4 S= methanol ]. A wide range of hydrogen bonds (of the O-H…O, N-H…O and N-H…N type) and π-π stacking interactions are present in the crystal structures.

**Conclusions:**

All compounds contain the dinuclear units, in which two Cu (II) ions are bridged by four *syn*, *syn*-η^1^:η^1^:μ carboxylates, showing a paddle-wheel cage type with a distorted octahedral geometry. The compounds **1** &**2** contain a single dimeric unit while **3** &**4** contain two dimeric units. The structures **3** and **4** are very interesting co-crystals of two paddle wheel molecules. Also it is interesting to note that the compounds **3** &**4** are isostructural with similar cell parameters. Both the compounds **3** &**4** differ in the solvent molecule coordinated to copper in one of the dimeric units. In all the four compounds, each of the copper dimers has an inversion centre. Every copper has a distorted octahedral centre, formed by four oxygen atoms (from different carboxylate) in the equatorial sites. The two axial positions are occupied by copper and the corresponding ligand.

## Background

**Figure 1:**
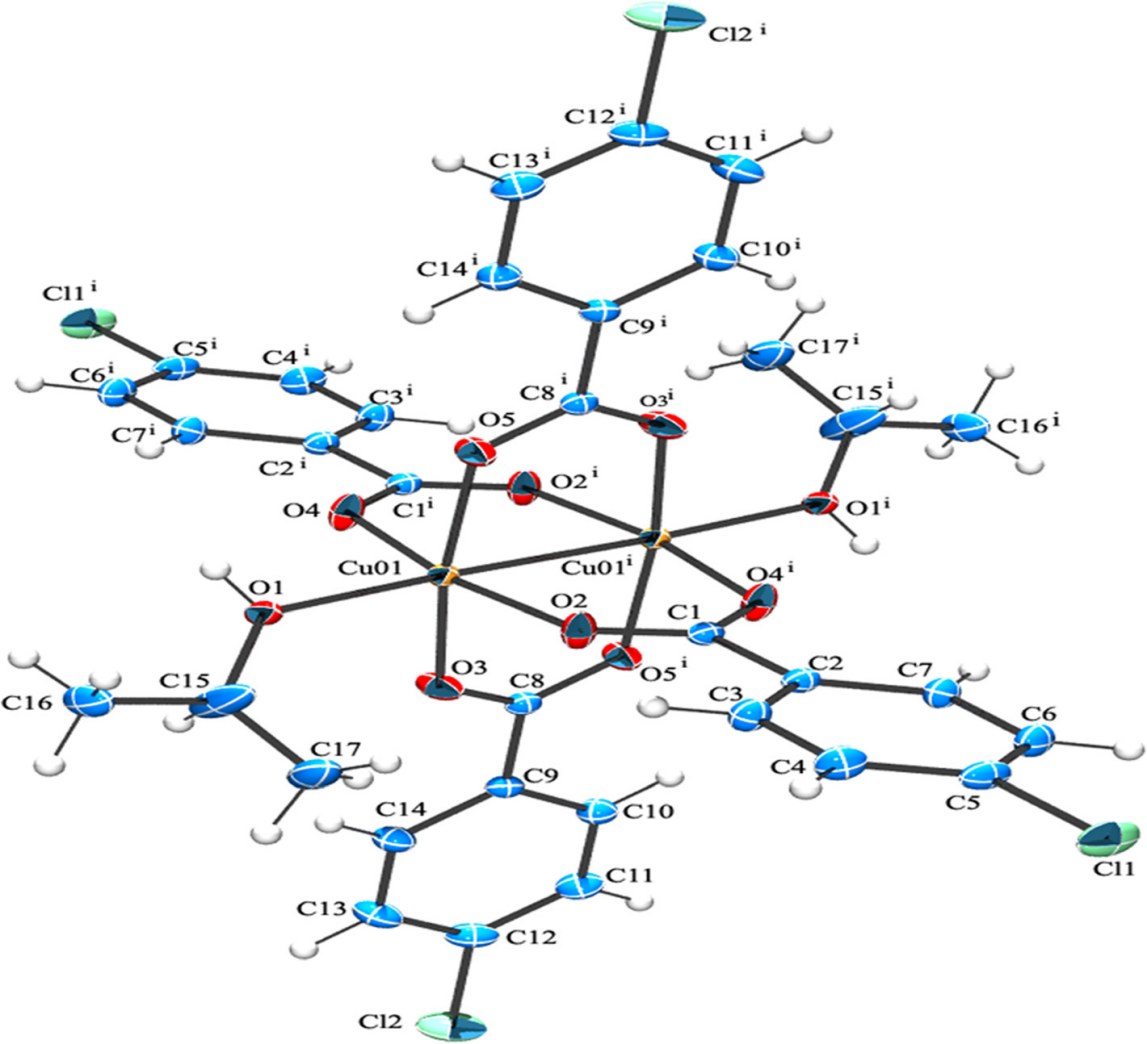
**ORTEP view of compound (1) showing the atom-numbering scheme.** Displacement ellipsoids are drawn at the 50% probability level and H atoms are shown as small spheres of arbitrary radii [symmetry code a: 1-x,1-y,1-z].

**Figure 2:**
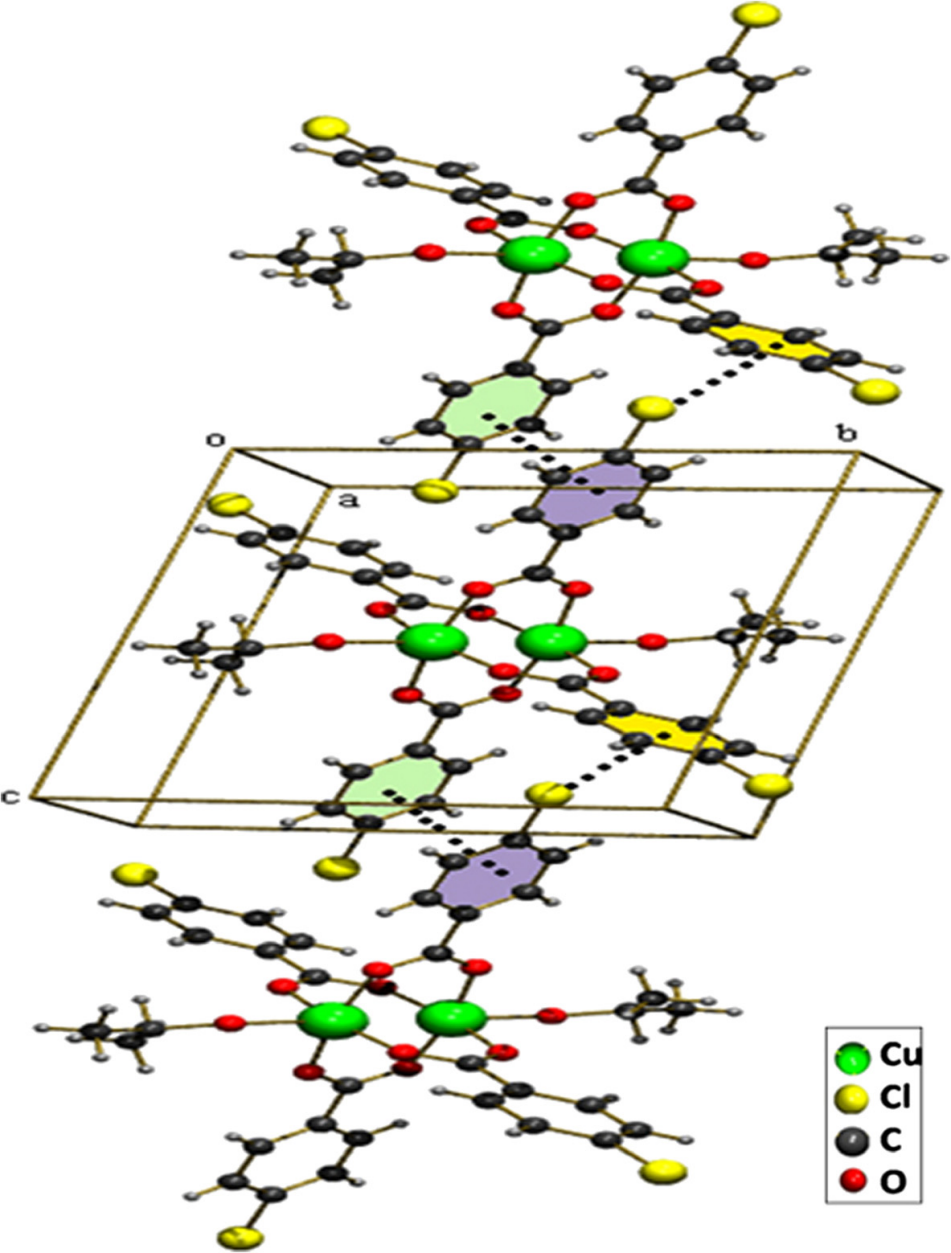
Formation of 2D sheet due to the π –π stacking and Cl - π interactions between 4-chloro benzoate molecules.

**Figure 3:**
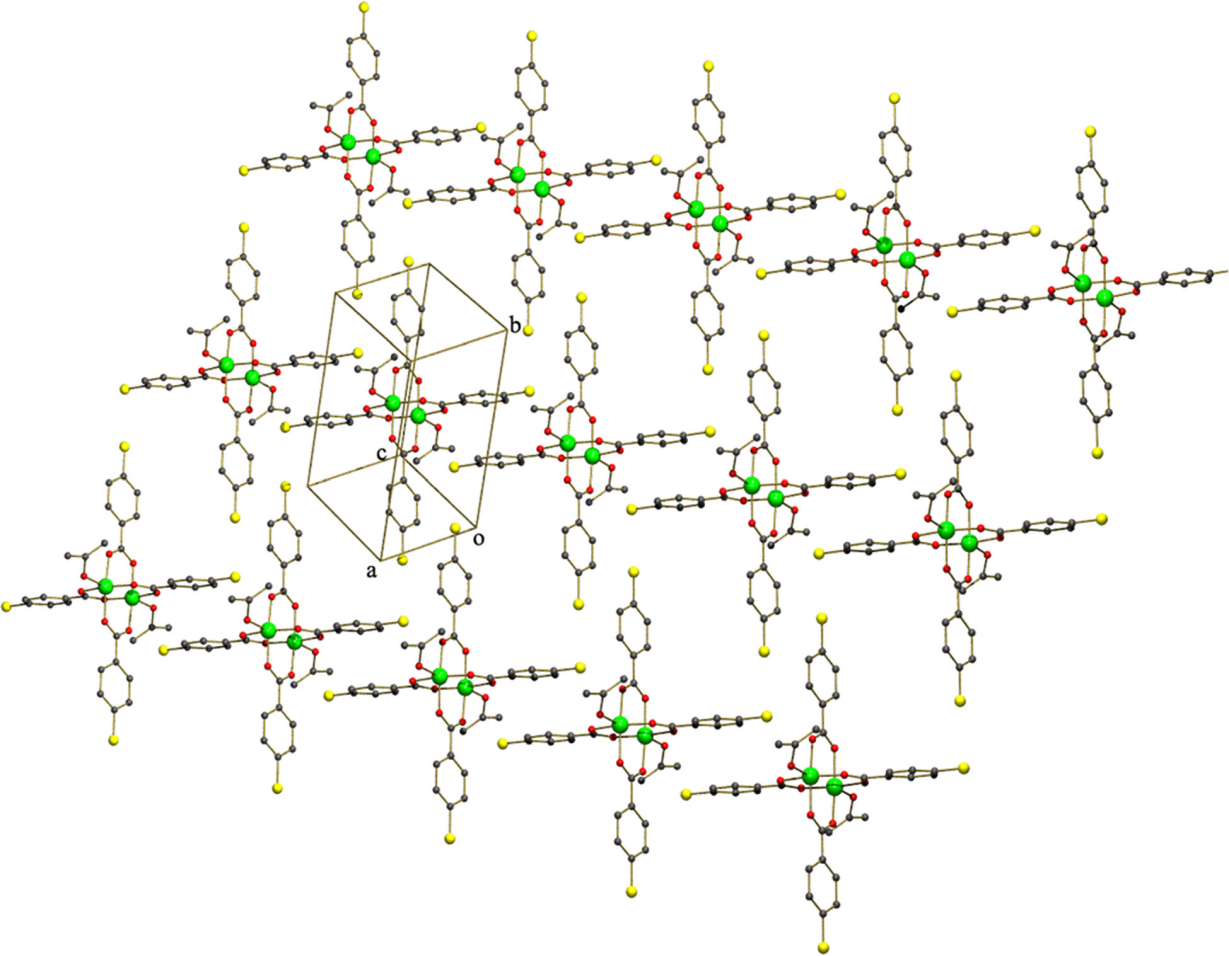
**The packing diagram and cyclic hydrogen bonded motif R**_**2**_^**2**^**(8) in (2).** Hydrogen atoms of the benzoate molecules have been omitted for clarity.

**Figure 4:**
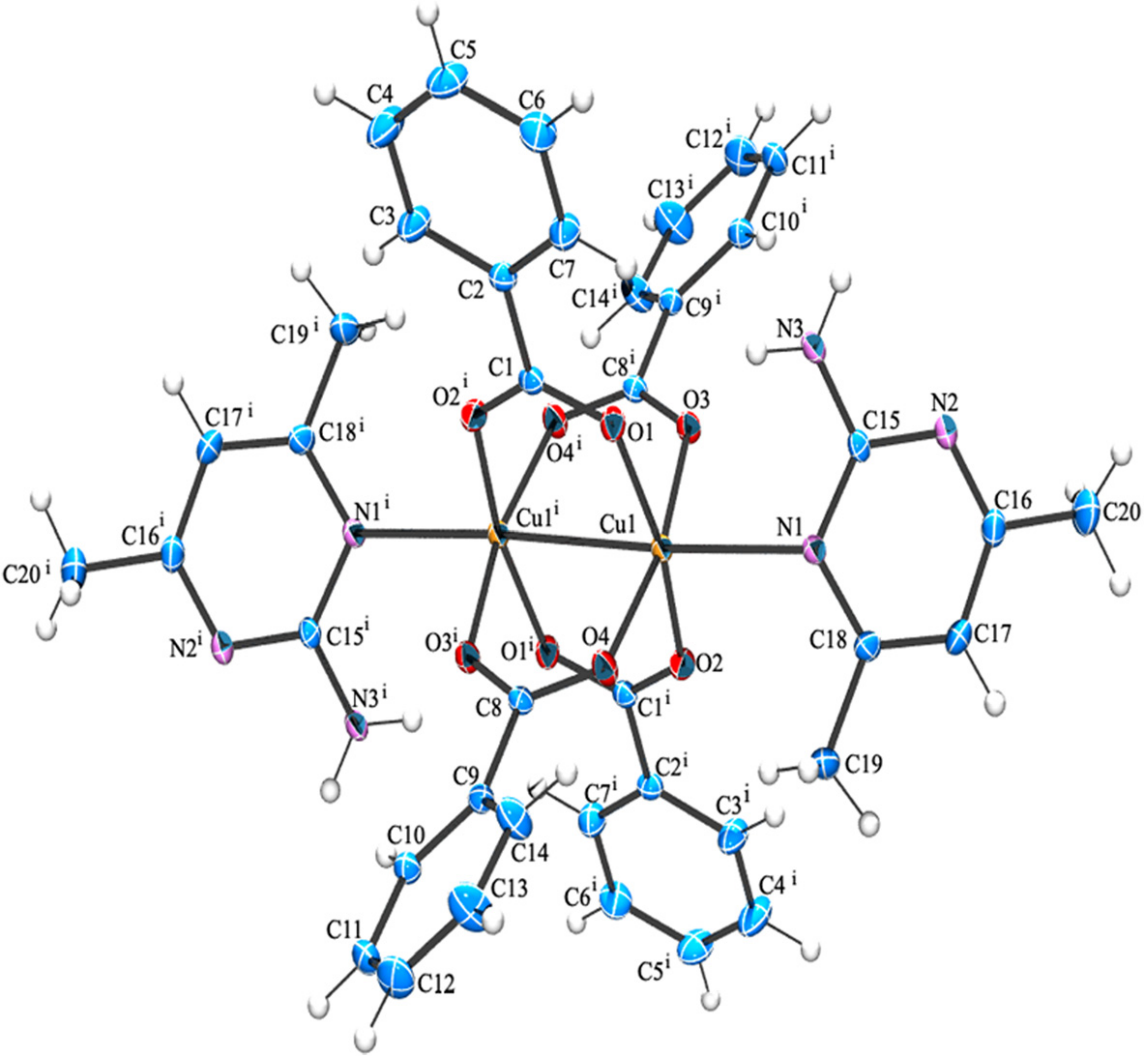
**ORTEP view of compound (3) showing the atom-numbering scheme.** Displacement ellipsoids are drawn at the 50% probability level and H atoms are shown as small spheres of arbitrary radii [symmetry code: a(-x,1-y,-z)] [symmetry code: b (1-x,-y,1-z)].

Copper carboxylate complexes have properties of importance in various areas and accordingly have been extensively studied [1]. They are known to form different kind of structures even with same kind of ligand. The reasons for this diverse nature are the basic nature of ligand, steric factor, starting compound, solvents etc. They also have properties of special interest in the fields of biology and magnetism [2]. Since the first copper(II) carboxylate dimer *ie* copper(II) acetate hydrate [Cu_2_(MeCO_2_)_4_(H_2_O)_2_] reported by van Niekerk and Schoening in 1953 [3], there have been reports of these kind of compounds. The dimeric copper (II) carboxylates [Cu_2_(RCOO)_4_ L_2_] are found to contain two or more antiferromagnetically coupled metal centres [4-7] and their magneto-structural correlation has been studied extensively [8]. Halogenated 2-thiophene carboxylic acids are used as building blocks for a new family of insecticides which also possess low mammalian toxicity [9]. The metal complexes formed by the interaction of thiophene carboxylic acid show an elevation in their biological activity [10,11]. It is also used in study of biological systems involving microbial degradation studies of sulphur containing compounds [12,13]. In contrast, in this regard, the use of carboxylic acid ligands with thiophene skeleton to construct Cu (II)– carboxylate compounds has been less investigated to date. Pyrimidine and aminopyrimidine derivatives are biologically very important compounds as they are components of nucleic acids [14]. The carboxylate group exhibits a variety of coordination behaviour displaying non identical bonding modes towards metal cations, such as monodentate and chelate, as well as η^1^:η^2^:μ^2^ bridging ligands in syn, syn, syn, anti, and anti,anti conformations [15,16]. In all the compounds **1**–**4** the syn-syn arrangement (Figures 1, 2, 3, 4) of carboxylate groups keeps the copper ions close enough making the possibility of copper-copper interaction [17]. The most important feature of the six fold coordinated copper complex is the close approach of the Cu-Cu distance [16,18]. In this frequently occurring octahedral geometry each of the copper atoms forms (in addition to the bonds from four oxygens that lies in the mean plane) a bond with copper atom and the corresponding axial ligand or solvent molecule. The co-crystals of inorganic complex with another inorganic complex are not common [19]. Recently there have been reports of co-crystal of two or more inorganic neutral complexes, there are also reports each neutral part having paddle wheel structure [19-22]. In this paper we report the synthesis and crystal structures of four copper (II) complexes with amino pyrimidine and different substituted carboxylate ligands which also include two isostructural, co-crystals of two inorganic neutral complexes.

## Results and discussion

### IR spectra

The FT-IR spectra of the complex **1**–**4** were recorded in a KBr pellet. The IR spectra of **1**–**4** gives the values of the asymmetric ν_asym_(COO^–^), symmetric ν_sym_(COO^–^) and Δν [the difference between (ν_asym_(COO^–^) and ν_sym_(COO^–^)]. This can indicate the coordination mode of the (COO^–^). Δν for compounds **1**–**4** are 183 cm^-1^, 208 cm^-1^, 162 cm^-1^, 162 cm^-1^ respectively. This result indicates the symmetrical bridging coordination mode of the carboxylate (syn, syn-η^1^:η^1^:μ^2^) giving rise to a paddle-wheel type structure [16,23-26]. For compounds **1, 3,** and **4** the difference was all less than 200 cm-1, which indicated the symmetrical bridging coordination mode of the carboxylate. However, the difference between ν_asym_ (COO-) and ν_sym_ (COO-) for compound **2** was greater than 200 cm-1. The hydrogen bond between N3-H6---O1 renders the symmetrical carboxylate asymmetrical, which is attributed to the large difference between ν_asym_(COO-) and ν_sym_(COO-) for compound **2**[27]. The IR spectra of **3** and **4** are essentially similar. In the IR spectra of **2**–**4**, ν_asym_(COO^–^) is split into two components, which is due to the hydrogen bonding of the bridged carboxylate ligand [28].

### Crystal structure

(1) $$\begin{array}{c} \mathrm{Crystal}\;\mathrm{structure}\;\mathrm{description}\;\mathrm{of}\\ \;\left[{\mathrm{Cu}}_{2}{\left({\mathrm{ClC}}_{6}H_{4}\mathrm{COO}\right)}_{4}{\left(\mathrm{Isopropanol}\right)}_{2}\right] \end{array}$$

**Figure 5:**
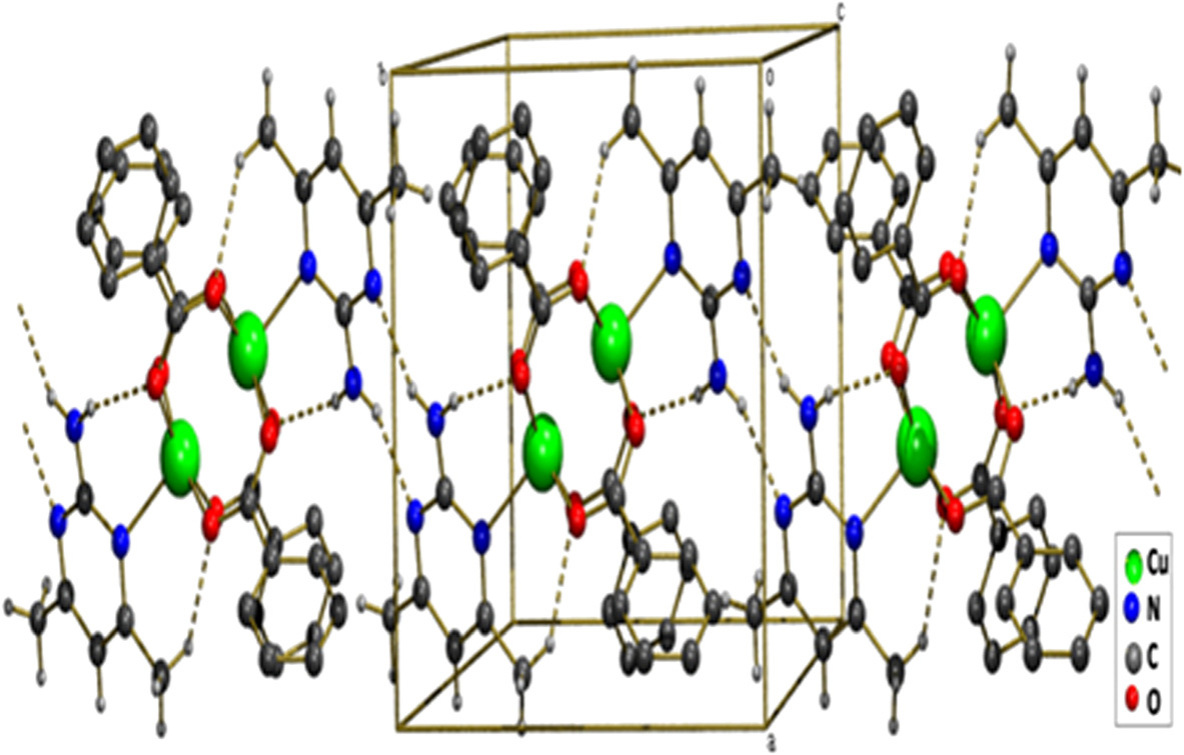
Showing the packing diagram of (1) and the π –π stacking and Cl - π interactions between 4-chloro benzoate molecules.

The title compound [Cu_2_(ClC_6_H_4_COO)_4_(Isopropanol)_2_], **1** was obtained by serendipity while trying to crystallize mixed ligand complex involving 2-Amino-4,6-dimethyl pyrimidine in isopropanol solvent. The molecular structure of **1** is more comparable with copper (II) acetate hydrate. Each copper atom is six coordinated in an octahedral fashion by four equatorial oxygen atoms belong to four carboxyl groups (Table 1) with Cu-O_eq_ distance which ranges from 1.959(2) to 1.965(2)Å. The O atoms at the apical positions belong to isopropanol. The Cu-Cu distance is small hence it is also considered as a bond. An ORTEP view of the dimer is shown in (Figure 1). π-π stacking interaction is found between two 4-chloro benzoate rings (C2, C3, C4, C5, C6, C7) - (Figure 5) along the a axis: d(cg-cg) 3.8105 Å. Cl - π interaction (Cl. . .cg distance,3.7701 Å) is found in between Cl of the 4-chloro benzoate ring and the six member ring comprised of (C9, C10, C11, C12, C13, C14) along the c axis (Figure 5). This leads to a 2D sheet (Figure 2).

(2) $$\mathrm{Crystal}\;\mathrm{structure}\;\mathrm{of}\left[{\mathrm{Cu}}_{2}{\left(C_{6}H_{5}\mathrm{COO}\right)}_{4}{\left(\mathrm{AMPY}\right)}_{2}\right]$$

**Table 1:** Representing the dimers in compounds (1–4)

**Ligands**	**1**	**2**	**3**		**4**	
	**Dimer**	**Dimer**	**Dimer (i)**	**Dimer ( ii)**	**Dimer ( i)**	**Dimer ( ii)**
RCOO	4-Chloro Benzoate	Benzoate	5-TPC	5-TPC	5-TPC	5-TPC
L	Isopropanol	AMPY		AMPY		AMPY
S			Ethanol		Methanol	

General formula for (1) and (2) Cu_2_(RCOO) _4_(L)_2_ , general formula for (3) and (4) Cu_2_(RCOO) _4_(S)_2_ Cu_2_(RCOO) _4_(L)_2_.

**Figure 6:**
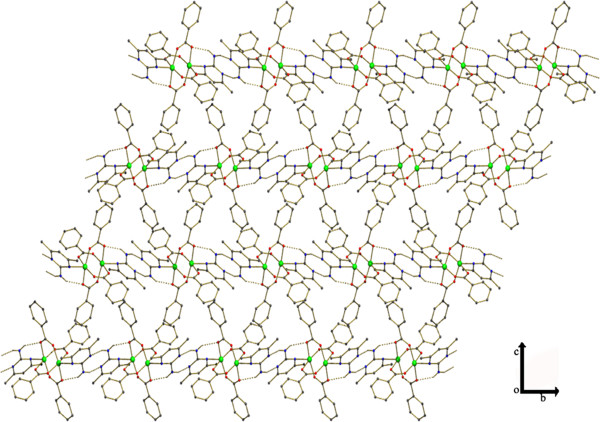
**ORTEP view of compound (2) showing the atom-numbering scheme.** Displacement ellipsoids are drawn at the 50% probability level and H atoms are shown as small spheres of arbitrary radii [symmetry code: a (1-x,1-y,-z)].

**Figure 7:**
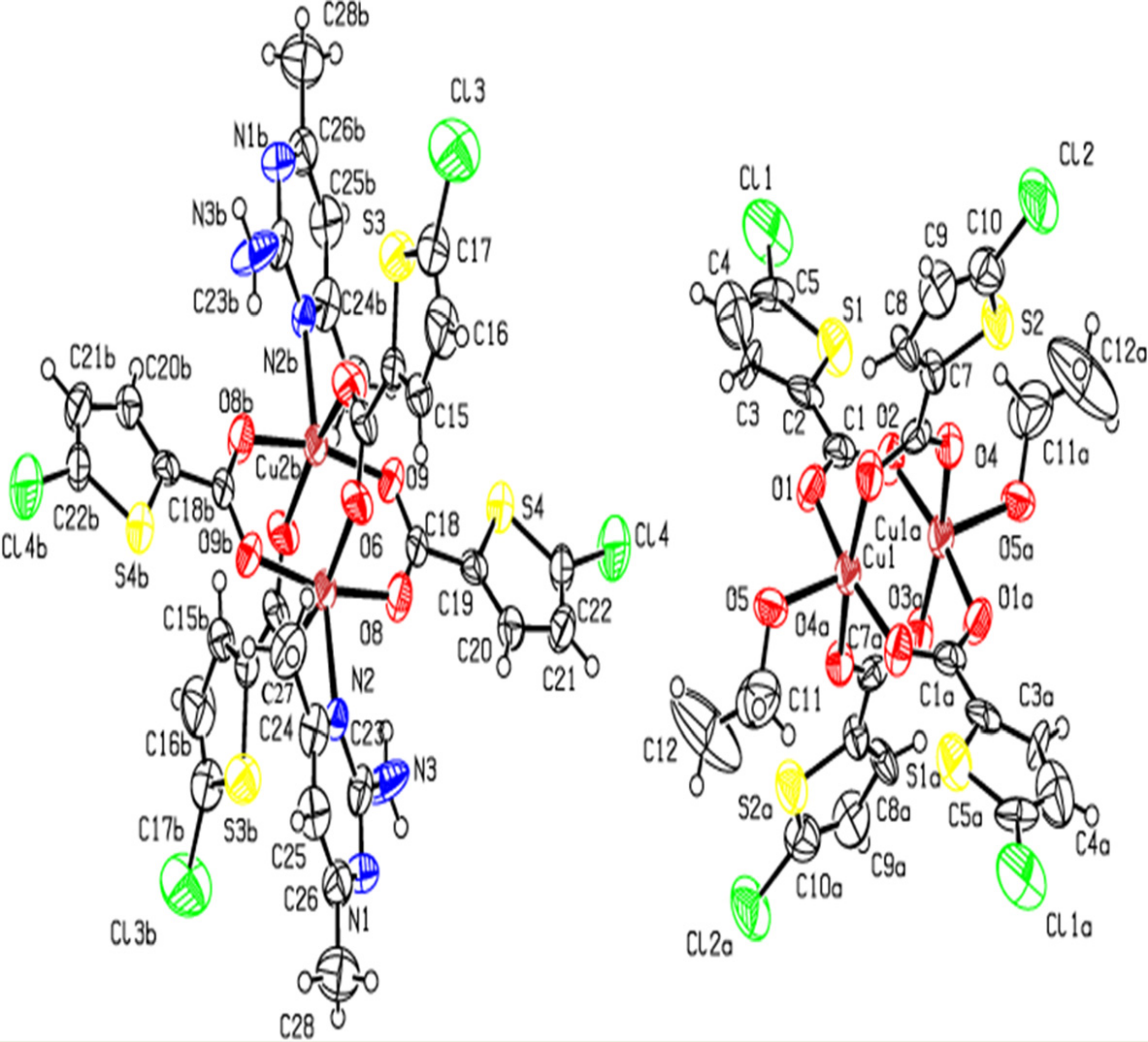
A view of supramolecular chains formed by N-H···O hydrogen bonds.

The structure consists of the centrosymmetric dimeric molecule [Cu_2_(C_6_H_5_COO)_4_(AMPY)_2_], (AMPY= 2-Amino-4,6-dimethyl pyrimidine) in which two copper(II) atoms are bridged *via* four benzoate anions thus forming a square base of four oxygen atoms (Table 1) around each copper (the average Cu–O distances lie from 1.9474(16) - 1.9655(16) Å). The apical positions of the octahedral coordination polyhedron are occupied by the N of a AMPY molecule (the Cu1 -N1 distance being 2.2724(18) Å and the Cu atom of the dimer). The Cu-N_ax_ distance in unsubstituted pyrimidine containing copper complexes lies in range of (2.033 (4) Å and 2.025 (4) Å) [29,30]. The copper atoms are displaced from the respective basal planes towards the apical nitrogen atoms by 0.176 Å, and the Cu–Cu separation within the dimer was found to be 2.6691(5) Å, is typical for the dinuclear paddle wheel type of copper coordination compounds. An ORTEP view of the dimer is shown in (Figure 6). The amino group of the axial ligand interacts with the pyrimidine nitrogen of the next molecule through a pair of N- - -H-N hydrogen bonds, forming a cyclic hydrogen bonded motif with graph set notation, R_2_^2^(8) (Figures 3, 7). The inter molecular N-H---N hydrogen bonds are found in between N3-H6a- - - N2 [symmetry code a: (1-x,1-y,-z)]. Intra molecular hydrogen bonds are found in between N3 -H6b- - -O1 [symmetry code b: (1/2-x,-1/2+y,1/2-z)] and C19 -H22c - - -O2 [symmetry code c: (-1/2+x,1/2-y,-1/2+z)].

(3) $$\begin{array}{c} \mathrm{Crystal}\;\mathrm{structure}\;\mathrm{of}\;\left[{\mathrm{Cu}}_{2}{\left(5-\mathrm{TPC}\right)}_{4}{\left(\mathrm{ethanol}\right)}_{2}\right]\\ \;\left[{\mathrm{Cu}}_{2}{\left(5-\mathrm{TPC}\right)}_{4}{\left(\mathrm{AMPY}\right)}_{2}\right] \end{array}$$

**Figure 8:**
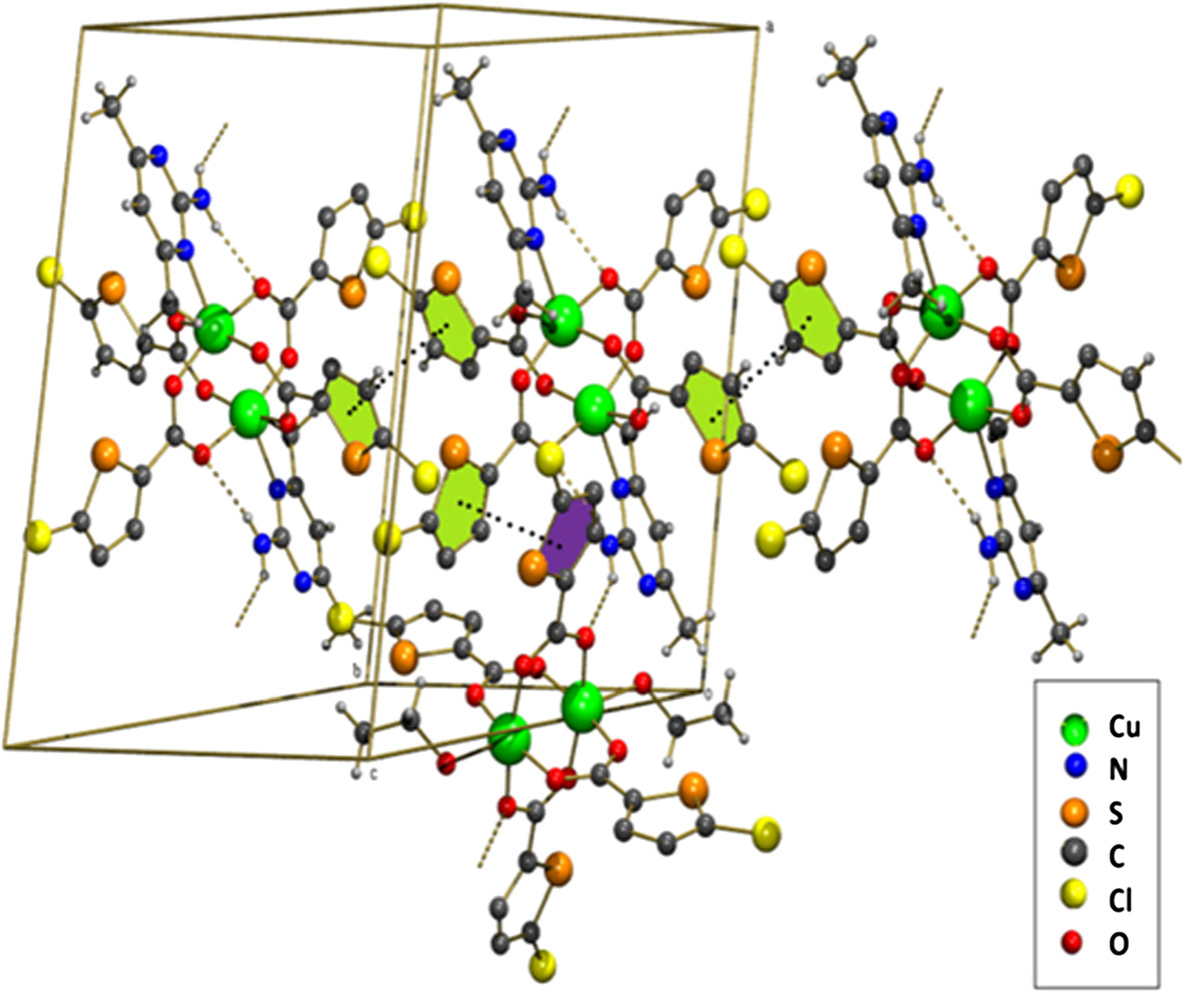
**The supramolecular architecture in compound (3).** The H atoms of 5-TPC rings are omitted for clarity.

**Figure 9:**
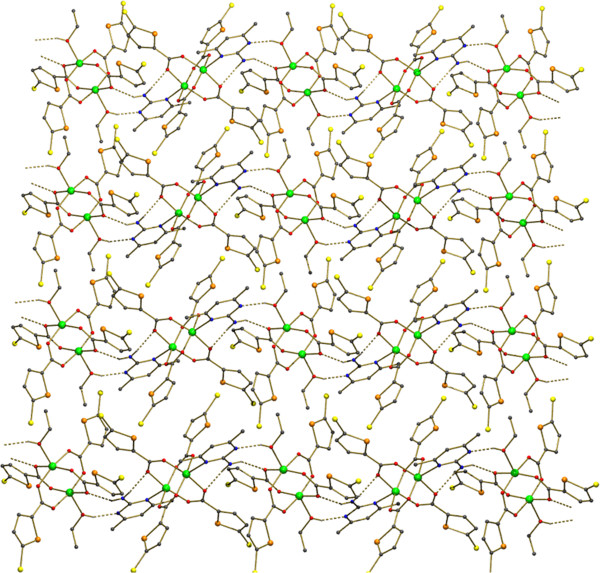
A view of 1D chains formed leading to a2D sheet in (3).

**Figure 10:**
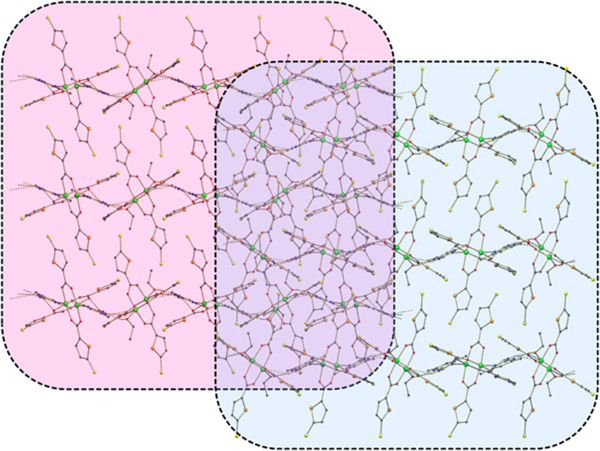
Two parallel 2D sheets stacked together in (3).

The compound **3** is a co-crystal and it contains two neutral inorganic units with the dimeric paddle wheel structure. The crystal structure of **3** consists of two centrosymmetric dimeric units [Cu_2_(5-TPC)_4_(ethanol)_2_] [Cu_2_(5-TPC)_4_(AMPY)_2_] (5-TPC = 5-Chloro-thiophene-2-carboxylate). In dimeric unit **(i)** two copper (II) atoms are bridged via four thiophene carboxylate anions while the apical positions are occupied by ethanol molecule and Cu atom of the same dimer (Table 1). In dimeric unit **(ii)** two copper (II) atoms are bridged via four thiophene carboxylate anions while the apical positions are occupied by AMPY molecule and Cu atom, thus forming a square base of four oxygen atoms around each copper. An ORTEP view of compound **3** is shown in (Figure 4). The average Cu–O distance in dimer **(i)** lies from 1.952(8) to 1.964(10) Å and the average Cu–O distance in dimer **(ii)** lies from 1.950(9) to 1.974(9) Å. The axial Cu-O distance in dimer **(i)** is 2.170(9) Å while the Cu-N distance is 2.311(11) Å in dimer **(ii)** is [less than that of **2**]. Intra molecular interactions are found in between N3-H3a- - -O8 and C27-H27a- - -O6 [symmetry code a: (-x, 1-y,-z)] (Figure 8). The dimeric unit (**i)** and (**ii)** are linked by N-H- - -O hydrogen bond between N3- H3b- - -O1 [symmetry code b:(1-x,-y,1-z)] (Figure 8). Additional stabilization is found through π-π stacking interactions between nearly parallel coordinated 5-TPC five member rings [S(4),C(19),C(20),C(21),C(22) & S(1),C(2),C(3), C(4),C(5)]: d(cg-cg) 3.798(7) Å. Another π-π stacking interactions are found between [S(3),C(14),C(15),C(16),C(17) & S(3),C(14),C(15),C(16),C(17)]: d(cg-cg) 3.693(9) Å. This N-H···O hydrogen bonds by the AMPY molecules lead to a 1D chain as in **2** leading to 2D sheets (Figure 9). Two of these sheets are stacked together by π-π stacking interactions (Figure 10).

(4) $$\begin{array}{c} \mathrm{Crystal}\;\mathrm{structure}\;\mathrm{of}\;\left[{\mathrm{Cu}}_{2}{\left(5-\mathrm{TPC}\right)}_{4}{\left(\mathrm{methanol}\right)}_{2}\right]\\ \;\left[{\mathrm{Cu}}_{2}{\left(5-\mathrm{TPC}\right)}_{4}{\left(\mathrm{AMPY}\right)}_{2}\right] \end{array}$$

**Figure 11:**
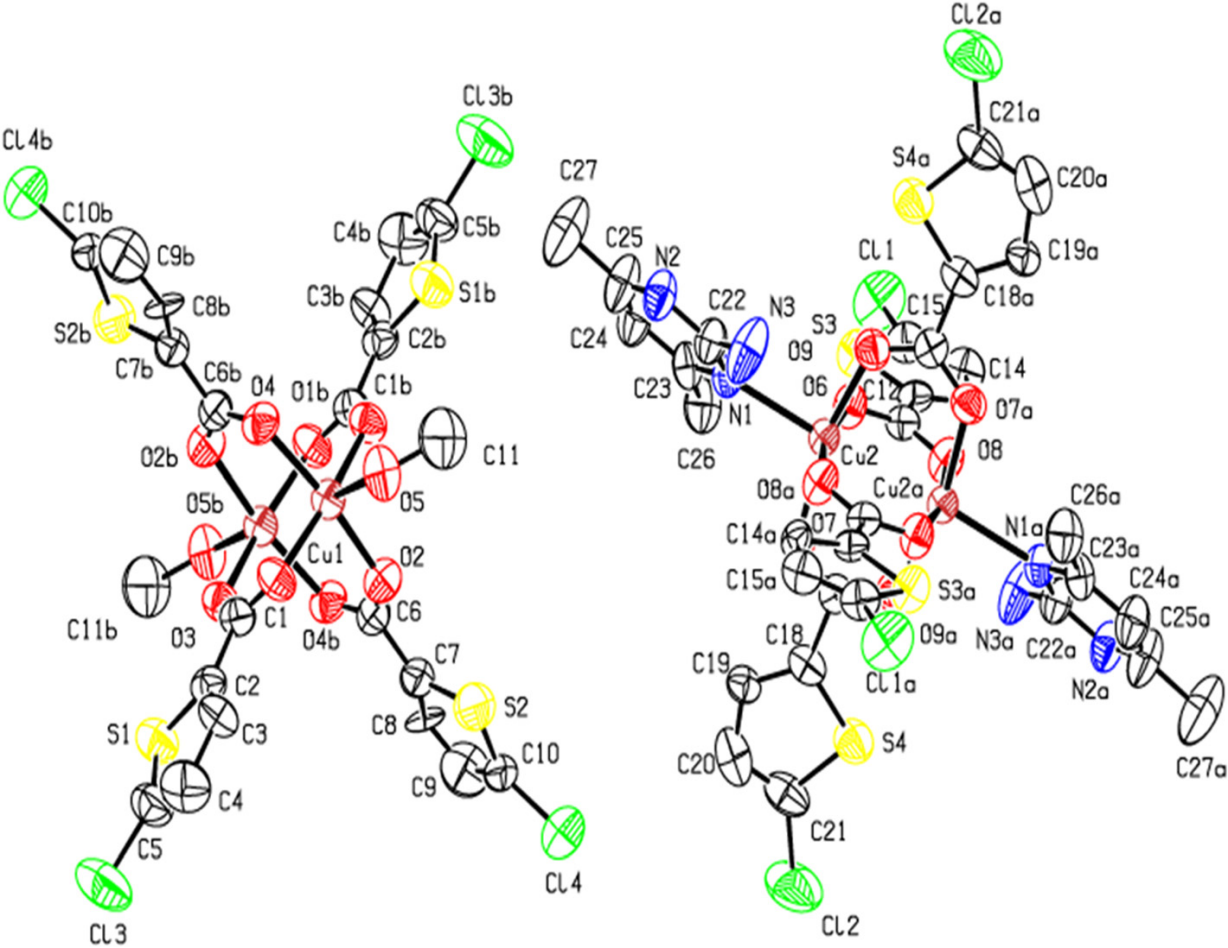
**ORTEP view of compound (4) showing the atom-numbering scheme.** Displacement ellipsoids are drawn at the 50% probability level and H atoms are omitted for clarity [symmetry code a:-x,1-y,1-z,] [symmetry code b:1-x,2-y,-z].

**Figure 12:**
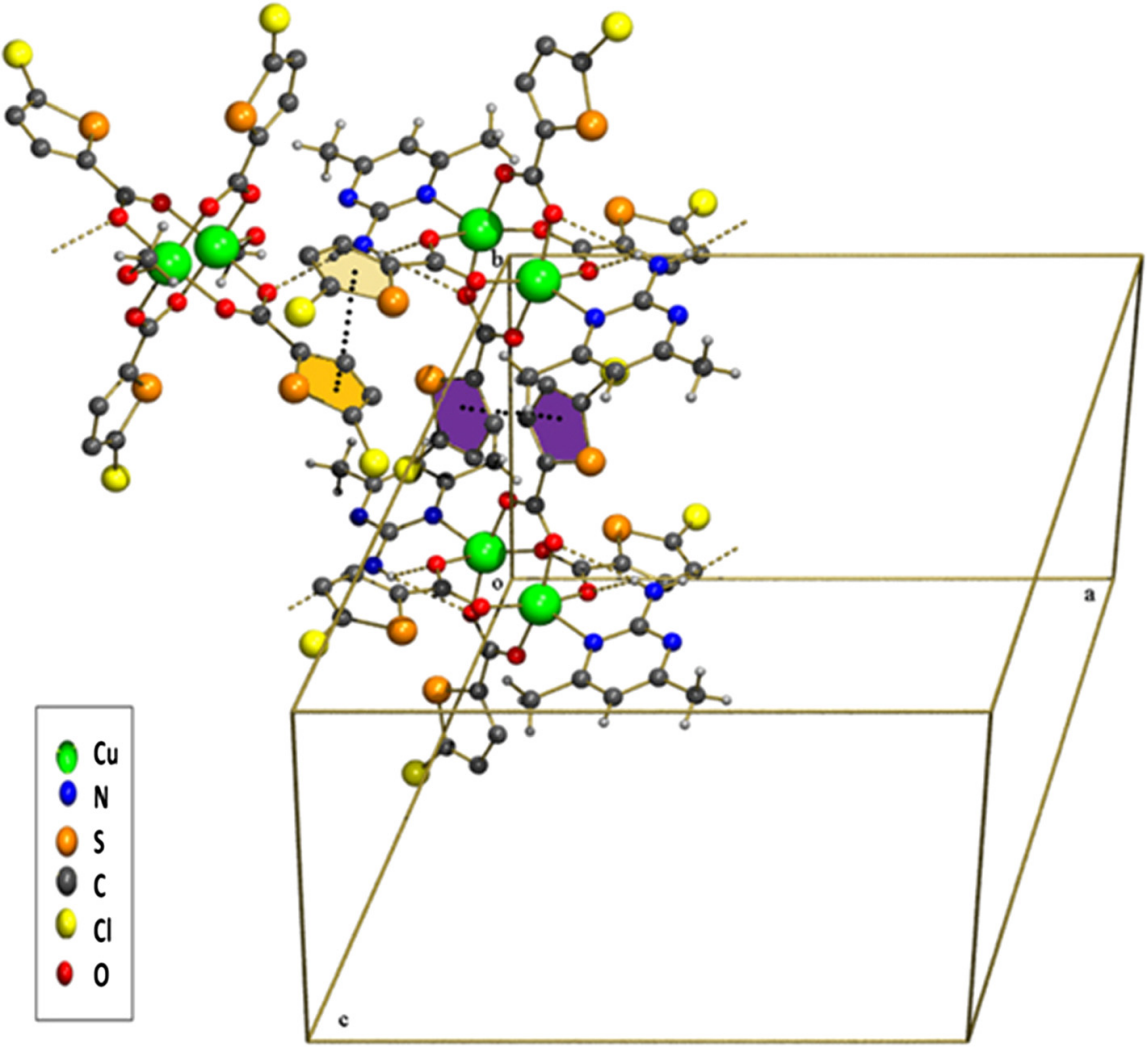
**A view of packing diagram (4) and the π-π stacking interactions between the two nearly parallel 5-TPC rings.** Hydrogen of 5-TPC are removed for clarity.

As compound **3** , compound **4** is also a co-crystal and it contains two neutral inorganic units with the dimeric paddle wheel structure. Compounds **3** and **4** are isostructural. Different from **3**, complex **4** exhibits a methanol solvent coordinated to copper ion in the place of ethanol. In the compound **4** there are two dimeric units with an inversion centre located between each of the copper ions [Cu_2_(5-TPC)_4_(methanol)_2_] [Cu_2_(5-TPC)_4_(AMPY)_2_]. In dimeric unit (**i)** two copper (II) atoms are bridged via four thiophene carboxylate anions while the apical positions are occupied by methanol molecule and Cu atom of the same dimer. In dimeric unit (**ii)** two copper (II) atoms are bridged via four thiophene carboxylate anions while the apical positions are occupied by AMPY molecule (Table 1) and Cu atom of the same dimer, thus forming a square base of four oxygen atoms around each copper. An ORTEP view of the structure is shown in (Figure 11). The average Cu–O distance in dimer **(i)** is 1.948(7) - 1.980(6)Å and the average Cu–O distance in dimer **(ii)** is 1.959(6)- 1.974(6)Å. The axial Cu-O distance 2.155(6) Å in dimer **(i)** is slightly less than that compared to **3**, while the Cu-N distance 2.301(7) Å in dimer **(ii)** is lesser than as compared to **2** and **3**. The C-H. . .O hydrogen bonds are observed inbetween C26 -H26a- - -O7 [symmetry code a:-x,1-y,1-z ]. The H3b attached to the NH_2_ of the AMPY is involved in bifurcated hydrogen bonding N3-H3b- - -O8 [symmetry code b:1-x,2-y,-z ] and N3 -H3b- - -O9 [symmetry code b:1-x,2-y,-z ]. The dimers **(i)** and **(ii)** are linked by N3-H3a- - -O4 hydrogen bond [symmetry code a:-x,1-y,1-z] (Figure 12). Two π-π stacking are observed between nearly parallel coordinated 5-TPC five member rings [S(3),C(13), C(14),C(15), C(16 and S(2),C(7),C(8),C(9),C(10)]: d(cg-cg) 3.801(2) Å. π-π stacking are observed between nearly parallel coordinated 5-TPC five member rings [S(4),C(18),C(19),C(20),C(21) and S(4),C(18),C(19),C(20),C(21)]: d(cg-cg) 3.668(6) Å. A view of the packing is shown in (Figure 12).

### Comparison of geometrical parameters

There is a relationship between Cu---Cu distance and the plane from which the Cu moves from the mean square base made up of four oxygen of the benzoate. It has been proposed after analysis of various dimeric Copper (II) acetate dihydrate analogue complexes [1]. As there is increase in Cu- - -Cu distance within the dimer, there is greater deviation of the Cu atom from the mean O4 plane .It has also been studied that whenever there are substituent’s on the 2-and/or 6-position of the axial pyridine ligand, the Cu-N distance is relatively long [31]. Based on these and extending these ideas to pyrimidine ligands we have tabulated the corresponding bond lengths and bond angles for all copper complexes (Table 2). In the dimeric units of **3** and **4** it can be noted that where ever there is an axial pyrimidine ligand, there is higher deviation of copper from the mean plane. Correspondingly we can note from the (Table 2) that whenever the Cu- - -Cu distance is higher, greater is the deviation of copper from the mean plane. From the (Table 2) it can be found that the Cu-N distance is high compared to that of Cu-N distances in similar systems containing un substituted pyrimidine ligands [29,30]. The increase in Cu…Cu distance and Cu-N distance in systems containing substituted pyrimidine ligands **3,4** may be due to steric and electronic factor of the apical ligand. It is found that such position of the ligand is very important as it strongly influences the Cu–Cu distances and also the carboxylate cage. The geometry around each Cu(II) ion can be best described as a slightly distorted octahedron showing tetracarboxylate type Cu_2_-(RCO_2_)_4_ unit. The Cu–Cu distance in compounds **1**–**4** lies in the range of (2.6143(5)- 2.758(3)Å), which is observed within the normal range for dinuclear paddle-wheel units in the structures of Cu(II) carboxylate complexes [16,32-38]. The axial N-Cu-Cu-N distance and O-Cu-Cu-O distances; are almost same as that of (Cu-Cu + Cu-N) and (Cu-Cu + Cu-O) distances respectively [Table 2]. This shows that the shortening of Cu-Cu distance is compensated by the axial Cu-N or Cu-O elongation. Thus we can conclude that there is an inversely proportional relationship between Cu–Cu and the Cu–L bond lengths, respectively. In copper carboxylate dimers if the Cu–Cu bond is longer shorter will be Cu–L distance and vice versa.

**Table 2:** Comparison of geometric parameters (Å) for some copper(II) benzoate dimers of compounds 1-4

		**Equtorial O-Cu-O distance (Å)**	**Axial Cu-O distance (Å)**	**Axial Cu-N distance (Å)**	**Cu - deviation from mean plane (Å)**	**Cu-Cu distance (Å)**	**O-Cu-O equatorial bond angle (°)**	**O- Cu-O axial bond angle (°)**	**O-Cu-N axial bond angle (°)**	**Cu-Cu + Cu-N (Å)**	**Cu-Cu + Cu-O (Å)**
Compound (1)		1.959(2) to 1.965(2)	2.167(2)		0.161	2.6143(5)	88.46(9)-92.98(9)	95.30(8)- 96.42(8)			4.7813
Compound (2)		1.9474(16) to 1.9655(16)		2.2724(18)	0.176	2.6691(5)	87.47(6)- 89.87(6)		91.47(6) - 101.43(7)	4.9415	
Compound (3)	Dimer (i)	1.952(8) to 1.964(10)	2.170(9)		0.160	2.658(3)	88.2(4)- 90.9(4)	95.0(4)- 96.8(4)			4.828
	Dimer (ii)	1.950(9) to 1.974(9)		2.311(11)	0.208	2.758(3)	87.9(4)- 89.4(4)		93.9(4)- 101.0(4)	5.0690	
Compound (4)	Dimer (i)	1.948(7) to 1.980(6)	2.155(6)		0.167	2.653(2)	88.0(3)- 91.5(3)	93.9(3)- 98.4(3)			4.808
	Dimer (ii)	1.959(6) to 1.974(6)		2.301(7)	0.207	2.7473(19)	88.1(3)- 89.1(3)		93.6(3)- 101.4(3)	5.0483	

## Experimental

### Materials and methods

Commercial starting materials were used without further purification. 5-Chloro thiophene 2- carboxylic acid (Hoechst Aktiengesellschaft), methanol and Cu(NO)_3_.6H_2_O (Qualigens, India) and ethanol (Changhhu Yangyuan chemicals, China) were used. IR spectra of the complex in region 400–4000 cm^-1^ were recorded as pressed disks (1% by weight in KBr) on a Shimadzu FT IR spectrophotometer.

(5) $$\mathrm{Synthesis}\;\mathrm{of}\;\left[{\mathrm{Cu}}_{2}{\left({\mathrm{ClC}}_{6}H_{4}\mathrm{COO}\right)}_{4}{\left(\mathrm{Isopropanol}\right)}_{2}\right]$$

**Scheme 1:**
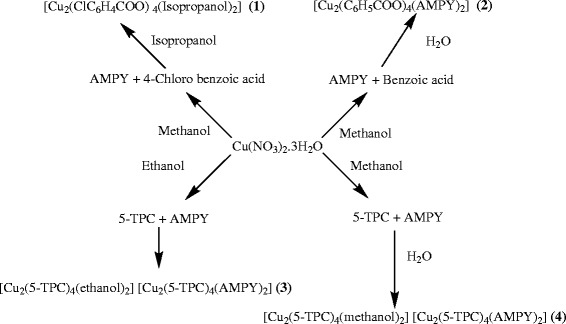
Preparation of compounds (1–4).

Cu(NO_3_)_2_.3H_2_O (0.0352 g) was dissolved in methanol (25 mL). To this solution, 4- chloro benzoic acid (0.0391 g,) was added and the mixture was stirred for 10 min to obtain a greenish blue solution. 2-Amino-4,6-dimethyl pyrimidine AMPY (0.0391 g) was added directly into the reaction mixture (Scheme 1). The resulting greenish solution was kept for crystallisation. After 72 hours a green precipitate appeared. The precipitate was recrystallised with 20 mL of (1:1) isopropanol/H_2_O mixture. After 2 days green crystals suitable X-ray diffraction studies were obtained.

(6) $$\mathrm{Synthesis}\;\mathrm{of}\left[{\mathrm{Cu}}_{2}{\left(C_{6}H_{5}\mathrm{COO}\right)}_{4}{\left(\mathrm{AMPY}\right)}_{2}\right]$$

A solution of Cu(NO_3_)_2_.3H_2_O (0.0302 g) in 15 mL Methanol was stirred over a hot plate magnetic stirrer for half an hour and benzoic acid solution (0.03053 g) dissolved in 10 mL of hot water was added to it. The mixture was stirred for additional 2 hours. A green coloured solution was formed. About (0.03079 g) of (AMPY) was dissolved in 10 mL of hot water and added to the reaction mixture. The mixture was stirred for 3 hours (Scheme 1). The resulting bluish green solution was kept for slow evaporation. Crystals were deposited at room temperature from the saturated solution. After 3 days green coloured crystals suitable for X-ray diffraction were obtained. The crystals were filtered and washed with small portions of methanol and were dried in air. The same product was obtained when the CuSO_4_.5H_2_O was used, but the yield was very low.

(7) $$\begin{array}{c} \mathrm{Synthesis}\;\mathrm{of}\;\left[{\mathrm{Cu}}_{2}{\left(5-\mathrm{TPC}\right)}_{4}{\left(\mathrm{methanol}\right)}_{2}\right]\\ \;\left[{\mathrm{Cu}}_{2}{\left(5-\mathrm{TPC}\right)}_{4}{\left(\mathrm{AMPY}\right)}_{2}\right] \end{array}$$

Cu(NO_3_)_2_.3H_2_O (0.0352 g) was dissolved in methanol (25 mL). To this solution, 5-Chloro-thiophene-2-carboxylic acid 5-TPC (0.0866 g) was added and the mixture was stirred and 10 mL H_2_O was added to obtain a green solution. AMPY (0.03079 g) was added directly into the reaction mixture (Scheme 1). After two days, crystals were deposited at room temperature from the saturated solution.

(8) $$\begin{array}{c} \mathrm{Synthesis}\;\mathrm{of}\;\left[{\mathrm{Cu}}_{2}{\left(5-\mathrm{TPC}\right)}_{4}{\left(\mathrm{ethanol}\right)}_{2}\right]\\ \;\left[{\mathrm{Cu}}_{2}{\left(5-\mathrm{TPC}\right)}_{4}{\left(\mathrm{AMPY}\right)}_{2}\right] \end{array}$$

The structure of compound **4** inspired us to design the preparation of compound **3**. For the preparation of **3** the procedure similar to that of **4** was followed, but ethanol was used in the place of methanol.

### Single crystal X-ray structure analysis

Intensity data sets were collected at room temperature, on a BRUKER SMART APEXII CCD [39] area-detector diffractometer equipped with graphite monochromated Mo Kα radiation (λ = 0.71073 Å). The data were reduced by using the program SAINT [39] and empirical absorption corrections were done by using the SADABS [39]. The structures were solved by direct methods using SHELXS-97 [40] and subsequent Fourier analyses, refined anisotropically by full-matrix least-squares method using SHELXL-97 [40] within the WINGX suite of software, based on F^2^ with all reflections. All carbon hydrogens were positioned geometrically and refined by a riding model with Uiso1.2 times that of attached atoms. All non H atoms were refined anisotropically. The hydrogens attached to the neutral alchol ligands (H1 in **1**, H5 in **3** and **4**) were located in difference Fourier maps and refined using O—H distance restraints of 0.82 (2) Å *via* the DFIX command. The molecular structure was drawn using the ORTEP-III [41] and POV-ray [42]. Crystal data parameters for compounds **1**–**4** were summarized in (Table 3). The crystals remained stable throughout the data collection.

**Table 3:** Crystallographic data for compounds (1–4)

	**Compound (1)**	**Compound ( 2)**	**Compound (3)**	**Compound (4)**
Empirical Formula	C_34_ H_30_ Cl_4_ Cu_2_ O_10_	C_40_ H_38_ Cu_2_ N_6_ O_8_	C_56_ H_44_ Cl_8_ Cu_4_ N_6_ O_18_ S_8_	C_54_ H_40_ Cl_8_ Cu_4_ N_6_ O_18_ S_8_
Formula weight	867.48	857.86	1883.33	1855.28
Temp, K	296	296	296	296
λ (Å)	0.71073	0.71073	0.71073	0.71073
Crystal system	Triclinic	Monoclinic	Monoclinic	Monoclinic
Space group	P -1	P 2_1_/n	P 2_1_/c	P 2_1_/c
a (Å)	6.6551(1)	10.1209(2)	16.8999(17)	16.8477(3)
b (Å)	11.5284(2)	11.3724(2)	11.8885(12)	11.8527(3)
c (Å)	12.4116(2)	17.1914(4)	19.118(2)	19.0258(4)
α(º)	94.814(1)	90	90	90
β (º)	103.696(1)	91.695(1)	105.533(7)	105.487(1)
γ (º)	100.183(1)	90	90	90
V (Å^3^)	902.69(3)	1977.85(7)	3700.8(7)	3661.33(14)
Z	1	2	2	2
ρ calcd (g/cm^3^)	1.596	1.441	1.690	1.683
μ (mm-1)	1.530	1.135	1.717	1.734
F(000)	440.0	884.0	1892.0	1860.0
Crystal size (mm)	0.15 × 0.12 × 0.10	0.20 × 0.18 × 0.15	0.09 × 0.06 × 0.05	0.16 × 0.15 × 0.12
No of reflections collected	5830	4880	3478	4049
Number restraints	1	0	1	1
Goodness-of-fit on F2	1.042	1.069	1.095	1.061
Final R1 index [I> 2σ(I)]	0.0463	0.0379	0.0651	0.0535
wR2 (all data)	0.1254	0.0973	0.1871	0.1725
Largest difference in peak and hole (e Å^-3^)	1.14 and -0.96	0.34 and -0.45	1.71 and -0.54	1.97 and -0.55

R_1_=∑(||Fo|-|Fc||)/∑| Fo |; *w*R_2_=[∑*w*(|F_o_|-|F_c_|^2^)^2^] / ∑*w*(|Fo|^2^)]^1/2^.

## Conclusions

We have crystallised four dinuclear paddle wheel copper(II) complexes of types Cu_2_(RCOO)_4_(L)_2_ and Cu_2_(RCOO) _4_(S)_2_ Cu_2_(RCOO)_4_(L)_2_, which retain the Copper acetate paddle wheel geometry. The compounds **1**–**4** contain different carboxylates, axial ligands, and solvents. The compounds **3** and **4** are co-crystals of inorganic complex with another inorganic complex which is not common. In addition to it **3** and **4** are differ only in the solvent molecule coordinated to copper atom. We have also investigated the supramolecular architectures present in the four compounds and compared their geometrical parameters. The structural results of compounds **1**–**4** are in good agreement with similar typed Cu(II) complexes with a paddle wheel geometry. The observed (Cu-Cu, Cu-O, Cu-N distances) are comparable with that of previously reported structures. The crystal structure of **1** is stabilised by π-π and C-H- - -π interactions. The crystal structure of **2** is stabilised by C-H---O and N-H---O interactions. The crystal structures of **3** and **4** are stabilised by C-H---O and N-H---O interactions and π-π interactions.

## Competing interests

The authors declare that they have no competing interests.

## Authors’ contributions

This work was prepared in the research group of PTM. He proposed the work and drafted the manuscript. SJJ participated in the design and presiding the experiments, collected the X-ray data and drafted the manuscript. Both authors read and approved the final manuscript.
